# Obesity and Food Insecurity at the Same Table: How Head Start Programs Respond

**DOI:** 10.5888/pcd9.110240

**Published:** 2012-07-26

**Authors:** Rachel A. Gooze, Cayce C. Hughes, Daniel M. Finkelstein, Robert C. Whitaker

**Affiliations:** Author Affiliations: Rachel A. Gooze, Cayce C. Hughes, Temple University, Philadelphia, Pennsylvania; Daniel M. Finkelstein, Mathematica Policy Research, Cambridge, Massachusetts. Mr Hughes is now at the University of Chicago, Department of Sociology, Chicago, Illinois, and Dr Finkelstein is now at Education Development Center, Waltham, Massachusetts.

## Abstract

**Introduction:**

Head Start is a federally funded early childhood education program that serves just over 900,000 US children, many of whom are at risk for obesity, are living in food insecure households, or both. The objective of this study was to describe Head Start practices related to assessing body mass index (BMI), addressing food insecurity, and determining portion sizes at meals.

**Methods:**

A survey was mailed in 2008 to all eligible Head Start programs (N = 1,810) as part of the Study of Healthy Activity and Eating Practices and Environments in Head Start. We describe program directors’ responses to questions about BMI, food insecurity, and portion sizes.

**Results:**

The response rate was 87% (N = 1,583). Nearly all programs (99.5%) reported obtaining height and weight data, 78% of programs calculated BMI for all children, and 50% of programs discussed height and weight measurements with all families. In 14% of programs, directors reported that staff often or very often saw children who did not seem to be getting enough to eat at home; 55% saw this sometimes, 26% rarely, and 5% never. Fifty-four percent of programs addressed perceived food insecurity by giving extra food to children and families. In 39% of programs, staff primarily decided what portion sizes children received at meals, and in 55% the children primarily decided on their own portions.

**Conclusion:**

Head Start programs should consider moving resources from assessing BMI to assessing household food security and providing training and technical assistance to help staff manage children’s portion sizes.

## Introduction

Head Start serves just over 900,000 low-income preschool-aged children through approximately 2,000 programs whose early childhood education services are supported by grants from the US Department of Health and Human Services (HHS) (1). Health promotion is part of Head Start’s holistic approach to children’s development (2), and programs allocate resources to optimizing children’s health and nutrition.

Among children entering Head Start, approximately one-third are overweight or obese (3). Furthermore, almost two-thirds are living in households with incomes at or below the federal poverty level (3), and approximately half of such US households are food insecure (4). On the basis of these data, 2 estimates can be made about children entering Head Start: 1) approximately one-third are living in food insecure households, and 2) approximately 10% are living in food insecure households and are either overweight or obese.

Whether food insecurity increases the risk for obesity in young children is unknown (5), but a mechanism by which food insecurity could be linked to obesity is that uncertainty about the availability of food may lead to overeating (6). For example, children arriving at Head Start from food insecure households may request or consume larger portions during meals and snacks. Many of these children may be overweight or obese. Therefore, regardless of whether food insecurity contributes to obesity, the co-occurrence of these conditions presents a potential challenge for Head Start staff as they try to provide a healthy eating environment for children.

Head Start programs are required to follow federal program performance standards. During meals, children and staff are required to “eat together family style and share the same menu to the extent possible” (7). Programs are also required to use funds from child nutrition programs funded by the US Department of Agriculture (USDA) to provide meals and snacks to children (7). The USDA’s Child and Adult Care Food Program (CACFP) provides most of the reimbursement to Head Start programs for food, and programs must abide by CACFP regulations regarding minimum portion sizes served (8). Programs are also required to assist families in obtaining a source of ongoing primary health care for their children (7). Despite existing regulations regarding health and nutrition, there are no specific requirements in 3 domains: 1) measuring or reporting children’s body mass index (BMI), 2) assessing and responding to household food insecurity, or 3) determining children’s maximum portion sizes by staff during meals. Using national data from the Study of Healthy Activity and Eating Practices and Environments in Head Start (SHAPES) (9-11), we describe program practices in these 3 related domains.

## Methods

### Survey development and administration

The overall purpose of SHAPES was to provide the first national description of obesity prevention practices in Head Start. The SHAPES survey was developed and administered in partnership with HHS and USDA, and the research protocol was approved by the institutional review board at Temple University. The average Head Start program contains approximately 6 centers, each with 50 to 60 preschool-aged children, and we administered the survey to every Head Start program director in the United States. In 3 prior reports, we presented details about the SHAPES survey and described various obesity prevention practices (9-11). This final report focuses on questions in 3 related domains: assessing BMI, addressing food insecurity, and determining portion sizes at meals.

Because the Head Start Program Performance Standards are intentionally broad (12) to allow for the variation in culture and resources where programs are implemented, the survey focused on more specific practices than those included in the standards. Survey questions pertaining to mealtime practices and portion sizes were adapted from an existing instrument (13), and original questions about BMI assessment and perceived food insecurity were developed for the SHAPES survey. The targeted survey respondents were program directors, who were encouraged to obtain assistance from their programs’ health and nutrition specialists when completing the survey. The survey was designed so that it could be completed in 30 minutes and would not require program staff to conduct any record reviews.

Administrative data and contact information for all Head Start programs were obtained from the Office of Head Start’s 2007 Program Information Report (14). The final sample of 1,810 Head Start programs excluded 80 programs total: 50 programs in US territories, 27 programs that did not provide direct services to children, and 3 that provided all services outside of centers. The 1,810 program directors were mailed a paper survey between February and April 2008.

### Survey items

Program directors were asked whether their program obtains information on children’s heights and weights and, if so, to indicate from a list of responses how their program obtained this information. We also asked whether their program uses height and weight measurements to calculate the BMI of children and whether the program staff discuss the height and weight measurements with families.

Food insecurity is defined as limited or uncertain availability of nutritionally adequate and safe foods or limited or uncertain ability to acquire food in socially acceptable ways (15). For young children, food insecurity refers to limited access to food in the household resulting from economic and social conditions. Children’s food insecurity is usually assessed by reports from parents or children’s primary caregivers and, therefore, could not be directly assessed in our survey of Head Start program directors. Nonetheless, we were interested in understanding from program directors how often their staff saw children who appeared to be food insecure and how the staff respond to this condition (16,17). To assess this, we asked “How often do staff in your program see a child who does not appear to be getting enough food to eat at home?” with 5 response options: very often, often, sometimes, rarely, or never. For purposes of this report, we use the term *food insecurity* to refer to the perception that a child does not appear to be getting enough food to eat at home.

We also asked program directors, “What do staff in your program do when they see a child who does not appear to be getting enough food to eat at home?” For this question, directors could mark all that applied from a list of options: 1) refer the family to the Special Supplemental Nutrition Program for Women, Infants, and Children (WIC), 2) refer the family to the Food Stamp Program (now called the Supplemental Nutrition Assistance Program [SNAP]), 3) refer the family to a food pantry or food bank, 4) keep additional food on hand to feed the child, 5) feed the child more on Mondays and Fridays, and 6) give food to the family to take home for the child. We categorized options 1 through 3 (making referrals) as indirect responses to food insecurity and options 4 through 6 as direct responses.

Program directors were asked to indicate from a list “which practice most closely describes how food is served to children during meals.” The response options involved combinations of 2 aspects of the practice: 1) whether “children serve themselves most foods” or “staff serve most foods to the children” and 2) whether “children mostly decide what size portions they take” or “staff mostly decide what size portions children may take.” The amount of food available for any child at a meal can be affected by the total amount of food available at the Head Start center or classroom for that given meal. Therefore, we also asked program directors to indicate from a list which practices they used to make sure there is enough food for everyone at meals.

### Data analysis

We describe the proportion of Head Start programs reporting a given practice in the 3 related areas (assessing BMI, addressing food insecurity, and determining portion sizes). We used χ^2^ tests to examine whether the proportion of programs reporting direct responses to food insecurity (providing food directly to children and families) differed by how often the program reported seeing children who appeared to be food insecure. We hypothesized that direct responses would be reported more commonly by programs in which the level of perceived food insecurity was higher.

## Results

Surveys were completed by 1,583 (87%) programs, which together enrolled 90% of all Head Start children. Nearly all program directors (99.5%) reported that they obtained height and weight information on all children in their program. Programs reported using various methods to obtain this information (Table 1), 74% of which listed more than 1 method. When program directors were asked to indicate the single most commonly used method for obtaining height and weight measurements, 70% reported that their staff members measured children’s heights and weights, and another 18% reported obtaining height and weight measurements from children’s health care providers (data not shown). Seventy-eight percent of programs reported calculating BMI for all children, 7% calculated BMI for some children, and the remaining 15% did not calculate BMI for any children. Nearly all program directors (98%) reported that their staff discussed the height and weight measurements with families; half of those directors reported that discussions took place with all families in their program, and the other half reported that they discussed height and weight measurements with only some families.

**Table 1:** Practices Used by Head Start Programs to Obtain Information on Children’s Height and Weight, US Head Start Programs, 2008 (N = 1,583)

Practice	%^a^
Head Start staff members measure the children’s heights and weights	89
Height and weight measurements are obtained from the child’s health care provider	70
Nurses from a school or school district measure the children’s heights and weights	23
Height and weight measurements are obtained from the child’s provider at WIC	30
Other	6

Abbreviation: WIC, Special Supplemental Nutrition Program for Women, Infants, and Children.

^a^ Percentages do not sum to 100% because program directors could mark all that applied.

Across all programs, 14% of directors reported that their staff very often or often saw children who appeared to be food insecure (did not appear to be getting enough food to eat at home); 55% reported that their staff saw this sometimes, 26% reported that their staff saw this rarely, and 5% reported that their staff never saw children who appeared food insecure. In those programs where staff saw children who appeared food insecure, 54% of program directors reported a direct response (providing food directly to children and families), and 98% of program directors reported an indirect response (referring families to community agencies for food assistance) (Table 2). Direct responses to perceived food insecurity were significantly more common in programs that reported a greater level of perceived food insecurity. For example, programs that often or very often saw children who appeared food insecure were twice as likely to report the practice of feeding the child more on Mondays and Fridays compared with programs that reported rarely seeing food insecure children.

**Table 2:** Program Responses to Perceived Food Insecurity in Children, US Head Start Programs, 2008 (N = 1,583)^a^

Type of Program Response to Food Insecurity	All Programs, %	Level of Perceived Food Insecurity^b^			*P* Value^c^

		Very Often or Often, %	Sometimes, %	Rarely, %	
**Direct**					
Keep additional food on hand to feed the child	37	46	38	32	.003
Feed the child more on Mondays and Fridays	21	33	20	16	<.001
Give food to the family to take home for the child	17	20	17	16	.49
Any direct response	54	66	54	49	<.001
**Indirect**					
Refer the family to WIC	94	96	94	92	.13
Refer the family to the Food Stamp Program^d^	89	92	89	88	.28
Refer the family to a food pantry or food bank	90	93	91	87	.03
Any indirect response	98	98	98	98	.97

Abbreviation: WIC, Special Supplemental Nutrition Program for Women, Infants, and Children.

^a^ Excludes 82 of the total 1,583 programs that reported never seeing food insecure children and 19 programs that did not respond to the question about perceived food insecurity or responses to food insecurity, for a total of 1,482 programs. Program directors could mark all that applied; therefore, percentages do not total 100%.

^b^ Program director report of how often staff saw children who did not appear to be getting enough food to eat at home.

^c^ *P* values calculated using χ^2^ test.

^d^ Now called the Supplemental Nutrition Assistance Program (SNAP).

Children’s portion sizes were determined differently among programs (Table 3). In 6% of programs, food arrived already portioned on each child’s plate; in 39% of programs, the staff primarily decided what portion sizes children received; and in 55% of programs, children primarily decided on their own portions. Children served themselves in most programs (79%), but in one-third the staff primarily decided what portion sizes children received. Directors reported various practices to ensure that enough food was available for all children at meals. Most of these practices required the staff to monitor children’s portions (Table 3).

**Table 3:** Practices for Determining Portion Sizes During Family-Style Meals, US Head Start Programs, 2008 (N = 1,583)

Practice	%
**Serving food to children during meals^a^**	
Children serve themselves most foods, and children mostly decide what size portions they take	53
Children serve themselves most foods, but staff mostly decide what size portions children may take	26
Staff serve most foods to the children, and staff mostly decide what size portions to give to the children	13
Staff serve most foods to the children, but staff mostly let the children decide what size portions they want	2
Food arrives already portioned on each child’s plate	6
**Making sure there is enough food to eat at meals^b^**	
Serving cups or utensils are provided that hold the amount of food that children should take	64
Staff pay close attention to make sure that children do not take too much	51
Staff tell children how much food to serve themselves	31
Staff serve the children to make sure there is enough food for everyone	14

^a^ n = 1,578 programs. Excludes 5 programs that did not respond to the question. Percentages were rounded to add to 100%.

^b^ n = 902 programs. Excludes 572 programs that reported that there was “usually more than enough food available,” 104 programs that reported that they did not need to ensure enough food to go around because meals arrived already portioned on each child’s plate, and 5 programs that did not respond to this question. Programs could mark more than 1 option; therefore, percentages do not total 100%.

## Discussion

This is the first national study to describe practices in Head Start programs in 3 related areas: assessing BMI, addressing food insecurity, and determining children’s portion sizes. Nearly all programs allocated staff time to the assessment of children’s height and weight, but not all calculated BMI or discussed children’s growth with families. Many programs reported that their staff saw children who appeared to not be getting enough to eat at home, and the programs often addressed this situation in ways that used the programs’ food resources, such as keeping extra food on hand to feed children, giving food to families to take home, and feeding children more on Mondays and Fridays. During family-style meals, a setting in which childhood obesity and food insecurity are at the same table, differing strategies were reported for how Head Start programs determined portion sizes. Some programs reported that teachers determined portion sizes, and other programs allowed children to decide how much food to take at meals.

Assessing BMI in schools has received increasing attention as an obesity prevention measure, but the practice remains controversial. There is no professional consensus regarding the usefulness of BMI screening in schools (18), and the practice has not been evaluated in early childhood education programs. Nearly all children attending Head Start are eligible for WIC, and the Program Performance Standards require programs to identify a source of primary health care for enrolled children. In addition to the practices of some Head Start programs, WIC program directors and other health care professionals also measure children’s height and weight and communicate the results to families.

For every child in a Head Start Program who does not appear to be getting enough food to eat at home, there is likely another child in the program who appears to be getting enough to eat at home but who is living in a food insecure household. Although obesity is often more visible than food insecurity, the latter has been associated with behavior and attention problems in preschool-aged children and with depressive symptoms and anxiety in mothers (19), all of which may affect children’s learning. We did not ask whether programs routinely screen families for household food insecurity, but such screening could be implemented as a routine part of the family assessment that is required at Head Start enrollment (7).

Evidence is increasing to support the role of serving appropriate portion sizes to prevent obesity in young children, but research is still needed to implement this practice in Head Start. For example, experimental studies show that mealtime energy intake of preschool-aged children increases when entree portion size is increased (20) and that children tend to select and consume less food if allowed to serve themselves (21). However, for children who are obese, food insecure, or both, more research is needed to determine what constitutes an appropriate portion size in any given meal or snack and how best to ensure that the child consumes that portion, especially at family-style meals. Therefore, we still lack evidence-based guidance for Head Start staff about how to determine children’s portion sizes and practice responsive feeding in circumstances where obesity and food insecurity are both present, sometimes in the same child (22).

Qualitative studies with Head Start teachers indicate that teachers face practical and emotional dilemmas during family-style meals when children in their classrooms have competing health and nutrition problems (23,24). Our finding that staff used several mechanisms to provide extra food to children who appeared hungry suggests that staff find the problem of perceived hunger emotionally compelling. However, 1 in 3 children entering a Head Start classroom each fall is overweight or obese (3), and Head Start has made an effort with its staff to prevent childhood obesity (25).

This study had several limitations. First, household food insecurity was not directly measured (26); however, the study was not designed to provide an estimate of the prevalence of food insecurity in Head Start. Second, the survey data reflected the perceptions of program directors and not those of the classroom teachers. Particularly in larger programs with many centers, the directors may have lacked accurate knowledge about the specific practices in question, and we did not attempt to validate the survey responses with on-site observations or record reviews. Third, we did not collect data on the types of foods actually eaten by children or the physical activity levels of children. Finally, the survey did not include questions about more detailed aspects of the reported practices, such as how height and weight were measured by staff or the content of discussions with parents about their children’s BMI.

Much like a family does, a Head Start program must allocate its limited financial resources to meet the varied needs of its children. More applied research is needed to assist Head Start programs in the optimal allocation of resources across the related areas of assessing BMI, addressing food insecurity, and determining children’s portion sizes. Awaiting such research, programs may consider the recommendations that follow.

Programs should re-evaluate whether they should directly measure children’s heights and weights, because the practice is resource intensive, may be redundant, and does not have clear utility. The practice is common, although it is not explicitly required by Head Start Program Performance Standards. Many Head Start programs are already closely coordinated with WIC clinics and pediatric offices, where BMI is assessed longitudinally. Furthermore, effective interventions are lacking for the treatment of obesity in preschool-aged children (27), and most approaches used to address obesity in Head Start are directed at all children, not just those who are obese (25).

Head Start should consider systematically assessing household food security. In contrast to BMI assessment, assessing food security requires no equipment and little training, is not systemically conducted in other settings, and identifies a condition that Head Start has the capability to address with its limited resources. A short, validated instrument exists to identify food insecure households (26), and this instrument is not regularly used in WIC or pediatric offices (28). Participation in programs such as SNAP and WIC may help to address food insecurity (29). Making referrals from Head Start to these food assistance programs may be important, because some families who are eligible for the programs may not be participating (3). In addition, families in Head Start may benefit from a greater collaboration with SNAP-Ed, the educational component of SNAP (30).

Finally, regardless of whether obesity and food insecurity are assessed in Head Start, these conditions will continue to exist at the same table and create challenges for staff during meals and snacks. Programs may wish to invest in more training and technical assistance to help their staff with managing children’s portion sizes at family-style meals, which would involve the use of such promising practices in this area (31) as the following: 1) allowing children to serve their own portions, 2) using child-sized dishes and utensils, and 3) encouraging children to select small first portions, then allowing children to request second portions.
